# Copy number variations among silkworms

**DOI:** 10.1186/1471-2164-15-251

**Published:** 2014-03-31

**Authors:** Qian Zhao, Min-Jin Han, Wei Sun, Ze Zhang

**Affiliations:** 1 Laboratory of Evolutionary and Functional Genomics, School of Life Sciences, Chongqing University, Chongqing 400044, China

## Abstract

**Background:**

Copy number variations (CNVs), which are important source for genetic and phenotypic variation, have been shown to be associated with disease as well as important QTLs, especially in domesticated animals. However, little is known about the CNVs in silkworm.

**Results:**

In this study, we have constructed the first CNVs map based on genome-wide analysis of CNVs in domesticated silkworm. Using next-generation sequencing as well as quantitative PCR (qPCR), we identified ~319 CNVs in total and almost half of them (~ 49%) were distributed on uncharacterized chromosome. The CNVs covered 10.8 Mb, which is about 2.3% of the entire silkworm genome. Furthermore, approximately 61% of CNVs directly overlapped with SDs in silkworm. The genes in CNVs are mainly related to reproduction, immunity, detoxification and signal recognition, which is consistent with the observations in mammals.

**Conclusions:**

An initial CNVs map for silkworm has been described in this study. And this map provides new information for genetic variations in silkworm. Furthermore, the silkworm CNVs may play important roles in reproduction, immunity, detoxification and signal recognition. This study provided insight into the evolution of the silkworm genome and an invaluable resource for insect genomics research.

## Background

Copy number variations (CNVs) are defined as DNA sequences ranging from 1 kb to few Mb that have different numbers of repeats among individuals [1,2]. Comparing with single nucleotide polymorphisms (SNPs), CNVs represent a higher percentage of genetic variation and have greater effects on a genome [3,4]. For example, CNVs play roles in determining phenotypic difference among individuals through changing gene structure and dosage, regulating gene expression and function [5-8]. In addition to normal phenotypic variation, CNVs are also related to genetic disease susceptibility [8,9]. And recently, CNV detection is substantially carried out in domesticated animals and these studies revealed that CNVs are associated with several phenotypic traits. For example, duplication of *KIT* gene in pigs determines the *Dominant white* locus [10]; while in sheep, the coat color is related to the duplication of *ASIP*[11]. In ridgeback dogs, hair ridge and predisposition to dermoid sinus are caused by duplication of 4 genes (*FGF3, FGF4, FGF19* and *ORAOV1*) [12]; and in Shar-Pei dogs, the wrinkled skin phenotype and a periodic fever syndrome are caused by upstream duplication of *HAS2*[13]. Also, partial deletion of *ED1* gene in bovine caused anhidrotic ecodermal dysplasia [14]. In avian species, CNV in intron 1 of the *SOX5* gene led to the pea-comb phenotype in chicken [15]. Thus, detection of CNVs at a whole-genome level can give a lot of useful information and has been carried out in several domesticated animals, including pigs, sheep, cattle, dogs,horses and chickens [16-28] as well as crops [29]. However, there is no information on CNVs in silkworm.

The domesticated silkworm (*Bombyx mori*), a model of Lepidoptera insects, has great economic value because of its silk production as well as its value as a good bioreactor [30]. It is widely accepted that *B. mori* is domesticated from the wild silkworm, *Bombyx mandarina,* about 5000 years ago [31]. And nowadays, more than 1,000 *Bombyx mori* inbred and mutant strains are kept all over the world [32]. In 2008, an estimated 432 Mb silkworm genome was published [33], with 8.5-fold sequence coverage and N50 size of ~3.7 Mb. And 87% of the scaffold sequences anchored to all 28 chromosomes, which can provide us a reliable genome to analyze the CNVs in silkworm. A previous study showed that the copy number of carotenoid-binding protein (*CBP*), a major determinant of cocoon color, varied greatly among *B. mori* strains [24]. Thus, the detection of CNVs at a whole-genome level is necessary for understanding phenotypic variations between different silkworms.

As far as we know, comparative genomic hybridization (CGH) and SNP arrays are routinely used for CNV identification [34-37]. However, the power of CNV detection is easily influenced by low probe density. In addition, although a subset of CNVs showed evidence of linkage disequilibrium with flanking SNPs [38], a significant number of CNVs located in the regions are not well recovered by SNP arrays [39,40].

With the development of next-generation sequencing (NGS) and complementary analysis program, there are some better approaches to screen CNVs systematically at a whole-genome level. Generally, NGS employed the read depth (RD) methods to analyze data and previous studies indicated that data with the genome coverage greater than 4 fold are sufficient for RD detection of CNVs [25,41-43]. To date, several methods have exploited sequence data in 1000 Genomes Project Pilot studies to detect CNVs [44,45]. And several programs are developed to analyze CNVs. These programs included CNAnorm ( http://www.precancer.leeds.ac.uk/), Bayesian information criterion [46], ReadDepth [47], CNV-seq [48], mrsFAST [49] and so on [50]. Specifically, an R package named readDepth can detect CNVs based on sequence depth and then invoke a circular binary segmentation algorithm to call segment boundaries [47]. This program has high sensitivity and specificity and is appropriate for screening CNVs in duplication and repeat-rich regions [47]. In this study, we resequenced 4 silkworms (2 domesticated silkworms and 2 wild silkworms). Then, we first used readDepth to screen the silkworm CNVs at a genome level and second used CNAnorm to recheck the CNVs, which can result in the high-confidence CNVs. Finally we tried to explore the distribution pattern and potential functions of the CNVs.

## Results and discussion

### Resequencing and CNV identification

We resequenced 4 silkworms: 2 domesticated and 2 wild silkworms. The sequencing coverage of these silkworms is greater than 5, indicating that the data are sufficient for CNV identification (Table 1, Additional file 1). The readDepth was employed to predict CNVs among four silkworms. The initial results of CNVs identified by readDepth were listed in Table 2 and the location information for each of initial CNVs is shown in Additional file 2. For further analysis, we retained only CNVs obtained by a more stringent criterion (RD differed significantly from the average of genome RD; see Methods). In order to prevent the false positive, we use this conservative filtering way, however, there should be some false negative regions that were abandoned from our analysis, especially regions with lower copy numbers in the genome. The filtration results are also listed in Table 2 (the detail information in Additional file 3). We identified ~348 suggestive CNVs, size ranging from 9.8 kbp to 34.5 kbp. The 348 CNVs covered 11.5 Mb. Then, we used another method CNAnorm to identify the CNV regions in silkworm. The potential CNVs identified by CNAnorm are listed in Additional file 4. Comparison of the results showed that 319 (10.8 Mb) of 348 CNVs by the readDepth were also identified by the CNAnorm (Additional file 4), which is about 2.3% of the silkworm genome. In the following analysis, we focused on these high-confidence CNVs (Additional file 5).

**Table 1:** Resequencing data of four silkworms

**Sample**	**Type**	**Raw bases**	**Valid bases**	**Average depth**	**Read STDEV**
N4	Domesticated	7788356400	6222459199	13.31	4.9
NanC	Wild	9567649400	7818574253	7.76	3
XiaF	Domesticated	9176146800	7302579044	14.42	6
AK	Wild	8745576000	7057560414	12.83	5.4

**Table 2:** The CNV calls in four silkworms

**Strain**	**Before filtering**			**After filtering by RD and CNAnorm**		
	**Total (bp)**	**Numbers**	**Average size (kb)**	**Total**	**Numbers**	**Average size (kb)**
N4	96546962	1082	89	4752536	150	32
XiaF	84976008	711	119	3337398	115	29
AK	190353801	640	297	3427899	89	39
NanC	84063418	433	194	2688123	60	45

The initial CNVs calls were kept if the RD values that differ significantly from the average RD and then filtered by CNAnorm.

Among four silkworms, the domesticated silkworm N4 contained the largest number of CNVs while wild silkworm NanC contained the fewest. As expected, the “uncharacterized chromosome” (ChrUn), sequences that cannot be mapped to the genome, contains most CNVs (~49%), which is consistent with the observation in cattle [22]. However, the CNVs on ChrUn need to be further investigated since ChrUn contigs are shorter and mapping of ChrUn sequence reads is ambiguous. In our study, CNV detection would be leveraged on the reference genome, thus, copy numbers are reported more like relative copies comparing to the reference genome. A well assembled reference as well as the well-annotated duplications in genome would be important to the CNV detection using this method. Therefore, the correct assemble of the contigs on ChrUn as well as annotations of repeats in the genome may help to improve the identification of CNVs. In order to get the accurate information about the CNVs and excluded false positives, clone-ordered-based approaches for sequence assembly and further annotation of repeats are needed in further study. The remaining CNVs are distributed on the silkworm chromosomes 1–27 and there is no CNV on the chromosome 28.

The positions of CNVs were determined independently within each silkworm and we compared them among different silkworms. Generally, we classified the duplicated sequences as shared or specific to an individual based on the predicted absolute copy numbers. The results showed that most of the CNVs were shared among two or more silkworms (Additional file 6). Specifically, the domesticated silkworm N4 had the largest number of unique CNVs while wild silkworm NanC contained the smallest number of unique CNVs (Table 2; Additional file 6). In general, a genome is assumed to be more tolerant to duplications than to deletions [51-53], accordingly, CNV gain should be more than loss. However, we found that silkworm had more CNV losses than gains, which is consistent with other species [16,17,19,23]. This result may be due to biological as well as technical reasons. One of the most important mechanisms which may be responsible for CNV formation, named as non-allelic homologous recombination, was proven to generate more deletions than duplications [54]. On the other hand, the detection method may favor the identification of deletions as reported in several other studies [20,44,55]. However, to validate the real status of CNVs, other techniques such as quantitative PCR (qPCR) is necessary.

**Figure 1:**
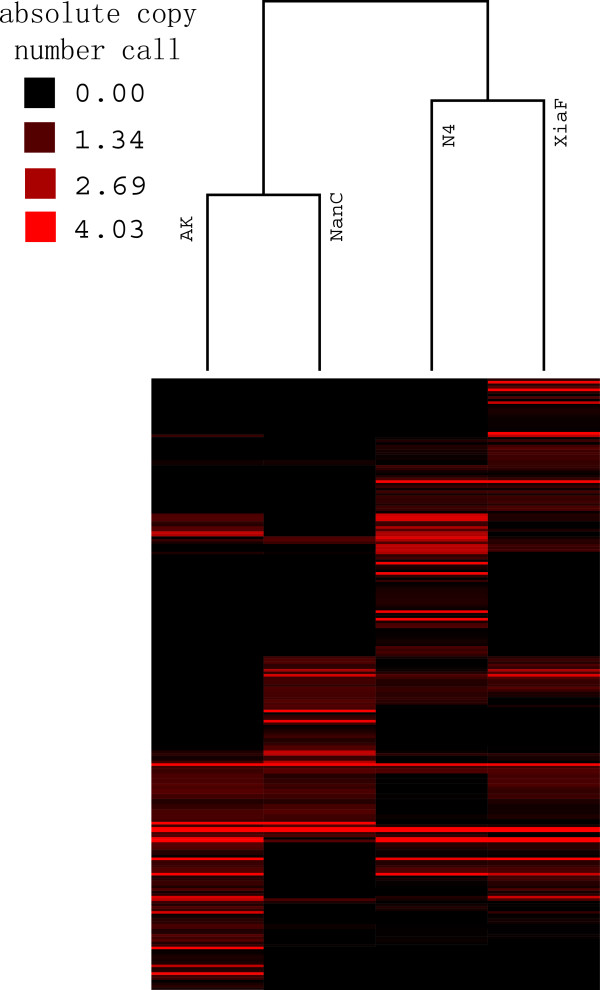
Cluster analysis of the 348 copy number variable regions in four silkworms.

As previous study showed, the heatmap can also reflect evolutionary relationships among diverse species [25]. Thus, we constructed a heatmap for 4 silkworms using absolute copy numbers in the CNV regions obtained by readDepth (Figure 1). As expected, 2 domesticated silkworms clustered together as other two wild silkworms did. A previous study suggested that a cluster tree constructed by the heatmap of individual-specific CNVs is usually consistent with the individual history [56]. Thus, genomic loci with great agriculture values or QTLs can be identified if there is a larger silkworm sample size and outgroup.

### Overlapping of CNVs with segmental duplications (SDs)

**Figure 2:**
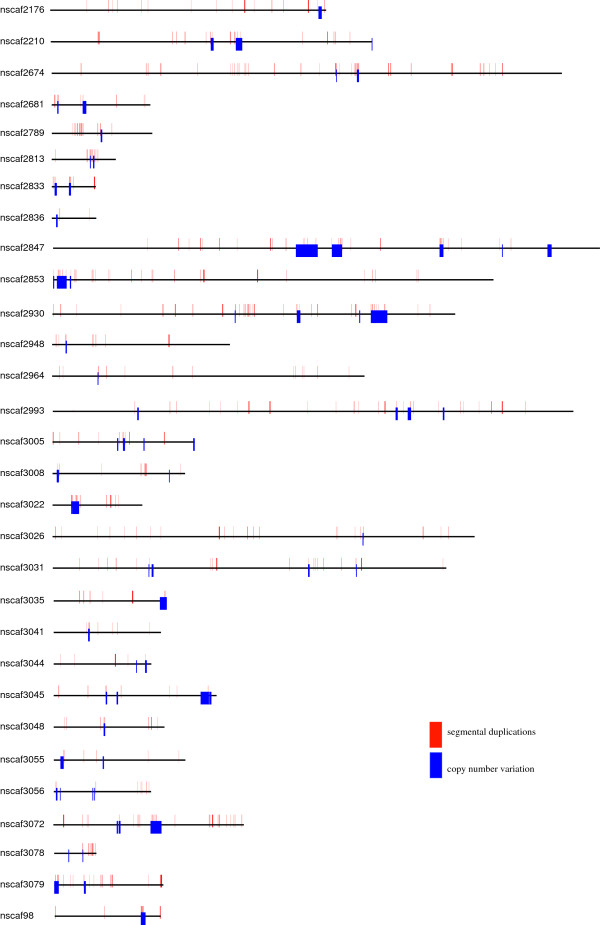
**Silkworm CNVs map.** Only 30 scaffolds were shown and all scaffolds with CNVs information were listed in Additional file 4. The silkworm assembly scaffold is represented as black bars. Larger bars in colors which intersect the scaffold represent the segmental duplications and copy number variation.

Previous studies showed that CNVs were enriched in SDs [1,2,57-61]. To test this, we compared the CNVs to the SDs identified by WSSD and WGAC approaches in our previous study [62]. Before filtering the initial CNVs using RD, there were about 94% of SDs exhibiting initial CNVs. And after filtration, approximately 60% of suggestive CNVs directly overlapped with SDs (Figure 2; Additional file 7).

Generally, it is accepted that SDs provide substrates of gene and genome innovation as well as genome rearrangement. SDs are also hotspots of formation of CNVs. Thus, SDs may arise from ancient CNVs fixed in the population [57,63-65]. As observed in other animals (dog, cattle, mouse, rat), there is a consistency (~50%-60%) between large CNVs and SDs (Figure 2) [16,22,60]. Thus, the association of large CNVs with SDs supports the hypothesis that CNV formation is mainly due to nonallelic homologous recombination (NAHR). This mechanism was proven to generate more deletions than duplications [54].

### Gene content of CNV regions and functional annotation

There are 208 functional genes resided at these high-confidence CNV loci. And 101 genes of them are duplicated in the silkworm genome. For example, CNV locus on scaffold 944 (scaffold 944: 6581–8724) encodes a HSP70 (heat shock protein 70) protein. In silkworm, a second copy of HSP70 is located on nscaf2801 (nscaf2801: 598000–599981).

**Figure 3:**
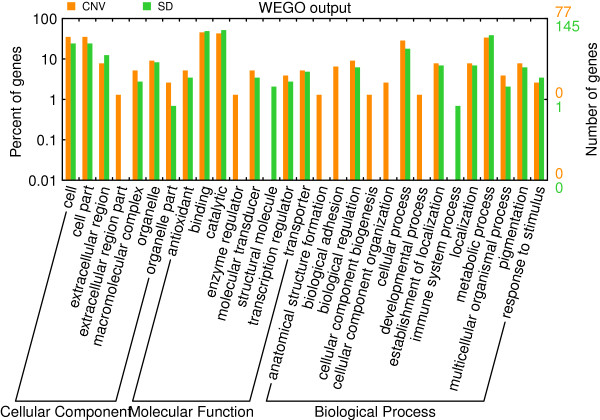
GO terms associated with the CNV regions and comparison with the genes in SDs.

We found that several genes in CNVs are involved in drug detoxification, defense and receptor and signal recognition, which is consistent with previous observations in mammals (human, mouse, cattle and dog) [16,20,58]. The expression patterns also validated this (Additional file 8). These gene families include Cytochrome P450, carboxylesterases, Moricin, Trypsin and olfactory receptor (Additional file 9), which shared similar GO terms (Figure 3). Interestingly, these gene families were repeatedly detected in CNVs of several mammalian genomes including humans, mouse, dog, cattle. This suggests that CNVs play important roles in evolution of organisms.

The functional genes located in CNVs possess a large spectrum of GO molecular functions (Figure 3) and provide a wonderful resource for validating the hypothesis that phenotypic variation within and among silkworms may be related to CNVs. For example, the carotenoid-binding protein (CBP), a major determinant of cocoon color, was found to have different copy numbers among the domesticated silkworms, ranging from 1 to 20 [24]. In present study, we also found that *CBP* gene (BGIBMGA009791-TA) is in CNV regions in 3 (XiaF, AK, NanC) of 4 silkworms investigated. This also further validated the efficacy of our CNV detection.

Genes with molecular function falling in binding and catalytic are enriched in the CNVs as well as SDs (Figure 3) (T-test, *p* < 0.01), which proved that particular gene classes are overrepresented in CNVs. A lot of these genes may very important in the lineage-specific adaptions of the organism to a particular environment. For example, Antimicrobial peptides (AMP) genes, which play important roles in innate immune system in insects [66], were found to be enriched in silkworm CNVs (6 genes were identified). Furthermore, since silkworm has to digest the secondary products in the mulberry leaves, some enzymes should be evolved to adapt to it [67]. For example, cytochrome P450 enzymes are involved in such biological processes in the silkworm [67]. In this study, we identified 10 genes belonged to P450 gene family. We also identified Carboxylesterase (COE), which involved in xenobiotic detoxification as well as pheromone degradation [68], in the CNVs regions. Other genes family related with important functions in lineage-specific evolution included Lipoprotein_11, heat shock proteins are also identified in our study (Additional file 9).

### Comparative analysis of silkworm CNVs

**Figure 4:**
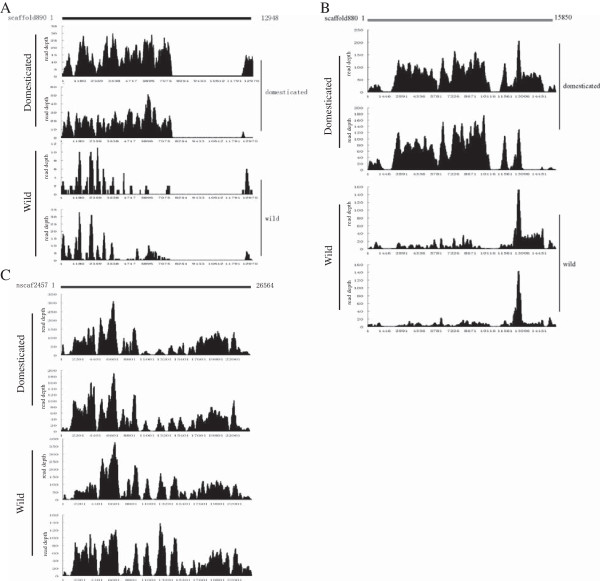
**Depth comparisons of CNVs in four silkworms.** The average read depth are listed in Table 1. The read depth are shown the RD for XiaF, N4, NanC and AK. **(A)** Depth comparison of CNVs in four silkworms for the region 1–12948 of scaffold 890 shows wild-specific region (loss). **(B)** Depth comparison of CNVs in four silkworms for the region 1–15850 of scaffold 880 shows domesticated-specific region (gain). **(C)** Depth comparison of CNVs in four silkworms for the region 1–26564 of nscaf 2457 shows all-possess region (gain).

In order to obtain information related to phenotypic characteristics as much as possible, we classified CNVs as individual-specific, domesticated-specific, wild-specific and all-possessed. Generally, most of the CNVs were shared among two or more silkworms (Additional file 6). However, we identified 80 individual-specific CNVs. Domesticated-specific CNVs are more than wild-specific ones (44 CNVs in domesticated vs. 36 CNVs in wild-specific). Furthermore, the read depth validated this result (Figure 4). Take scaffold 890 as example (Figure 4A), the RD for NanC is less than 4 comparing with the average depth of 7.76. And AK’ RD is less than 7 comparing with the average RD of 12.83.

We investigated the genes in the regions of domesticated-specific, wild-specific and all-possessed CNVs. The domesticated-specific CNVs contained 24 functional genes, while wild-specific CNVs contained only 17 genes. We also surveyed the functions and expression patterns of these genes. Most of the genes in these CNV regions are related to detoxification, reproduction and immunity since they were expressed in midgut, testis, ovary and homocyte, respectively. In domesticated-specific CNV regions, there is an extra gene cluster which was expressed in silkgland (Additional file 10). However, most members of this gene cluster were poorly annotated in the silkworm database, indicating that the functional information on the genes in CNVs has been very limited to date. This deserves further investigation in future.

### CNV validation by quantitative PCR

We used real time quantitative PCR (qPCR) to validate CNVs in 5 genomic regions as well as 10 genes. Four of five loci (genomic sequences) were validated by this method (Additional file 11). For the exception, the silkworm genome has two copies of Target_r1 (scaffold984:1…11044) based on the BLASTN searches against *B. mori*. And the qPCR results showed little variation among 4 silkworms (2 domesticated and 2 wild) at this locus. This might be: (1) prediction errors of CNVs, that is, the false positive; (2) polymorphisms such as indels and SNPs that influence binding of the qPCR primers. For four validated regions, we found that there was a big difference in copy number at the locus of Target_r3 between domesticated and wild silkworms. That is, domesticated silkworm contained more copies than wild type at this locus based on the qPCR results. Also, this region belongs to domesticated-specific region. Furthermore, we found that only one gene (BGIBMGA014594-TA) is located in this CNV region. However, this gene was poorly annotated so far. A previous study showed that this gene was specifically and highly expressed in testis, indicated that this gene may play important roles in reproduction [69]. Further study is needed to characterize its function.

**Figure 5:**
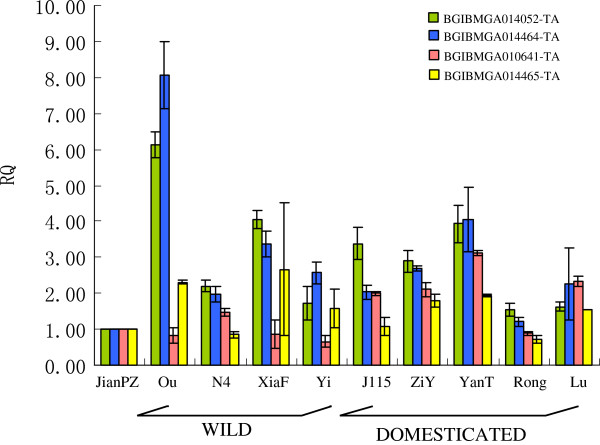
**qPCR confirmation.** Different bars represent different genes. X-axis shows the different individuals while Y-axis is the value of 2^-∆∆CT^ that is indicator of duplications (RQ). The domesticated silkworms include JianPZ, Ou, N4, XiaF, Yi, J115, wild silkworms include ZiY, YanT, Rong, Lu.

Besides, we also chose 10 genes to validate the presence of CNVs in different silkworms (Additional file 11). A total of 10 silkworms (4 wild silkworms and 6 domesticated silkworms) were examined: eight of ten genes can be validated by qPCR, except for two genes (BGIBMGA014051, BGIBMGA014594). *F*-test was performed to check whether copy number detected using qPCR showed homogeneity of variance between the reference silkworm and silkworms to be examined. The result suggested that all these 8 loci in silkworms to be examined had greater variance than those in the reference silkworm (*P* < 0.05) (Figure 5, Additional file 11), confirming that the CNVs identified in this study are reliable. For these 8 genes, one (BGIBMGA012385-TA) belonged to P450 gene family, one (BGIBMGA002901-TA) belonged to COesterase andone (BGIBMGA009791-TA) belonged to carotenoid-binding protein. A previous study of microarray expression profiling showed that two (BGIBMGA014464-TA and BGIBMGA014465-TA) of 8 genes were highly expressed in head, integument and hemocyte [69]. Another gene, BGIBMGA014052-TA, was specially and highly expressed in Malpighian tubule, implying its important role in detoxification in silkworm. BGIBMGA010640-TA, which is involved in lipid metabolic process (GO: 0006629), was highly expressed in midgut. Midgut of silkworm is very important because of its key functions in digesting, resistance and immune response. Genes expressed highly in midgut suggest its important roles in nutrient digestion and absorption, resistance and immune response in silkworm. A previous study used four pathogens to challenge silkworm and investigated the genome-wide gene expression profiles by a microarray [70]. We exploited this dataset to check the expression pattern of BGIBMGA010640-TA as well as expression patterns of another 7 genes that were proven to be resistant to nucleopolyhedrovirus (BmNPV) [71]. Like the above 7 genes, BGIBMGA010640-TA could be induced by 3 pathogens (Additional file 12) [70]. This suggested that BGIBMGA010640-TA may be involved in immune response of silkworm.

The CNVs (86.7%, 12/15) were confirmed to be positive CNVs by qRT-PCR (Figure 5, Addational file 8). It should be emphasized that not all true CNVs could be detected by qPCR, especially some low-copy duplications with less sequence similarities. Thus, 13.3% for false positive rate is a conserved estimate in our CNV analysis.

## Conclusion

We have constructed the first CNVs map in silkworm based on next-generation re-sequencing data. A total of ~319 CNVs were identified in the silkworm genome. We presented the frequency, pattern and gene-content of these CNVs. Our results indicated that the genes in CNVs may be involved in specific biological functions such as reproduction, immunity, detoxification and signal recognition. Besides, we identified 80 CNVs that may be individual-specific. Most of genes in these 80 regions were also related to reproduction or detoxification. The data presented in this study provided insight into the evolution of the silkworm genome and an invaluable resource for insect genomics research.

## Methods

### Data sets

#### Genome sequencing and read cleaning

Silkworm genome was obtained from previous studies [33,72]. We prepared libraries for four silkworms (two wild silkworms named as AK and NanC and two domesticated silkworms named as XiaF and N4). We sequenced them using Illumina (Hiseq2000) according to standard manufacturer protocols. The low-quality (Quality < 20) nucleotides were trimmed by sliding a 5 bp window.

#### Read alignment and CNV detection

We used the BWA program to align the paired-end reads to the silkworm genome reference [73], the criteria are the same as to previous study [47]. For the detection of CNVs among four silkworms, we have applied a program called readDepth [47] using a parameter 0.01 of an FDR rate, which resulted in bins with a size of 1.7 kbp. And readDepth calculates the thresholds for copy number gain and loss for each silkworm (Additional file 13). The readDepth uses a binning procedure to call copy number variants based on sequence depth and then call segment boundaries using a circular binary segmentation algorithm. Our previous results suggested that there are ~1.4% of SDs in the reference genome [62], which can help us to adjust the data in the program. The GC bias was corrected using LOESS method to fit a regression line to the data [41,47].

In order to find the high-confident CNVs, we calculated the read depth (RD) of the regions predicted by the readDepth. And we calculate the average read depth for the unique regions of silkworm identified before [62]. We only kept the regions with RD greater than 3 standard deviations from the mean [25]. Then, these regions whose RD differed significantly from the average of genome RD (Chi square test; *p* < 0.05) were termed as potential CNVs.

Because different algorithms can generate different CNV results [42], we used CNAnorm ( http://www.bioconductor.org/packages/release/bioc/html/CNAnorm.html) to recheck our CNV regions to reduce the false-positive or false-negative rate. We employed parameters of –readNum 150, −-saveTest, −-saveControl in PERL script of bam2windows.pl (a script in the CNAnorm package). The parameter lambda 7 was used to decrease noise without losing resolution and ploidy (ploidy = (sugg.ploidy(CNN4) + 1)) was used to check the potential CNVs in the genome.

#### Heatmap hierarchical cluster analysis

Heatmaps were obtained based on the absolute copy number call generated by readDepth. The gplots R package ( http://cran.r-project.org/web/packages/gplots/index.html) was employed to get the heatmap of the absolute copy number call in four silkworms.

#### Gene content analysis

Gene content of *B. mori* segmental duplications was assessed using the glean consensus gene set ( http://silkworm.genomics.org.cn/) [74]. We obtained a total of 14,623 silkworm peptides from SilkDB. In addition, using Gene Ontology (GO) [75], we tested the hypothesis that the molecular function, biological process, and pathway terms were under- or overrepresented in CNV regions. Furthermore, we compared the GO results between the genes from SDs and the genes from CNV regions. Pfam [76] was also used to annotate the function of the genes in CNV regions.

#### Quantification of CNVs in the silkworm genome by quantitative PCR

Genomics DNAs were extracted from domesticated and wild silkworms, and stored in Tris-EDTA (TE) buffer at 4°C. The primers used in qPCR are designed using Primer 5.0 and listed in Additional file 14. The principle for copy number quantifying using qPCR was described in previous study [77]. According to previous studies, *OR2* was chosen as control because of its highly-conserved sequence and single copy in the silkworm genome [24,78,79]. *Con_R* is a two-copy region in the silkworm genome according to *B. mori* genome database [71,72,80,81]. We also used this region as control to estimate copy numbers of target regions.

Each PCR reaction was prepared as follows: 10 μl of SYBR-Green PCR master mix, 1 μl of each primer (10 μM), 7 μl of water, and 1 μl of genome template. Quantitative real-time PCR was carried out using the ABI Stepone plus system. The thermocycler program had an initial 95°C denaturation step followed by 40 cycles consisting of a 10-s denaturation at 95°C, a 40-s annealing at 60°C, and a 30-s extension step at 72°C. At the end of each reaction, a disassociation curve was created, which was used to help to detect the presence of primer dimers of other unwanted amplification products that may produce a detectable cycle threshold (Ct) value. Copy number was analyzed according to comparative Ct method. The ∆CT and ∆∆CT were calculated by the formulas ∆CT = CT target – CT control (single copy) and ∆∆CT = ∆CT SD samples -∆CT single copy sample, respectively. The domesticated silkworm JianPZ was taken as a standard for determining gene copy number.

### Availability of supporting data

Raw sequence reads have been deposited in the ENA database (The European Bioinformatics Institute) with the accession number PRJEB5458 and can also be downloaded from http://bioinfor.cqu.edu.cn/read_silkworm/.

## Competing interests

The authors declare that they have no competing interests.

## Authors’ contributions

ZZ designed the study. QZ performed the analyses and experiments, and drafted the manuscript. MJH provided help in the data analysis and revised the manuscript. WS provided help in doing experiments and read the manuscript. ZZ supervised the study and revised the manuscript. All authors read and approved the final manuscript.

## Supplementary Material

Additional file 1 Basic information for RD and reads. Click here for file

Additional file 2 The initial results of CNVs identified by readDepth. Click here for file

Additional file 3 The suggestive ~348 CNVs. Click here for file

Additional file 4 The CNVs identified using CNAnorm. Click here for file

Additional file 5 The CNVs identified both by readDepth and CNAnorm. Click here for file

Additional file 6 Venn diagram showed the comparison of CNV content amongst different silkworms. Click here for file

Additional file 7 **Silkworm CNVs map.** The silkworm assembly scaffold is represented as black bars. Larger bars in colors which intersect the scaffold represent the segmental duplications and copy number variation. Click here for file

Additional file 8 **Expression profiles of the genes located in CNVs based on microarray data.** Hierarchical clustering with the average linkage method was performed. There were as many as 9 tissues used in the gene expression profiling. Click here for file

Additional file 9 **Functional annotation of genes located in CNVs.** Sheet1 shows the function predictions by BLAST search against nr database. Sheet2 shows the function prediction obtained by Pfam. Click here for file

Additional file 10 **Comparison of gene expression pattern located in domesticated-specific CNV regions and wild-specific CNVs based on microarray data.** Hierarchical clustering with the average linkage method was performed. There were as many as 9 tissues used in the gene expression profiling. The upper diagram showed the expression profiles of genes in wild-specific CNVs. Click here for file

Additional file 11 qPCR validation of predicted CNVs in silkworms. Click here for file

Additional file 12 **Expression profiles of 8 genes in silkworm challenged by four pathogens: *Bacillus bombyseptieus *(BB, gram-positive bacteria); *Beauveria bassiana *(BJ, fungus); *Escherichia coli *(EC, gram-negative bacteria); *B. mori Nuclear polyhedrosis viruses *(NPV, virus).** Data were collected from four time points (3 h, 6 h, 12 h and 24 h; for *Be. bassinan*: 6 h, 12 h, 24 h and 48 h) (Huang, 2010). Click here for file

Additional file 13 Thresholds for copy number gain and loss. Click here for file

Additional file 14 A list of primers used in qPCR. Click here for file
