# Supplementary material for: Copy number variations among silkworms
Source: BMC Genomics. 2014 Mar 31;15:251. doi: 10.1186/1471-2164-15-251 (PMC3997817; doi:10.1186/1471-2164-15-251)
Supplement: Additional file 1 — Basic information for RD and reads. [file 1471-2164-15-251-S1.docx]

**Additional file 1: Basic information for RD and reads.**

| Sample | Average depth | Coverage >=4X  (%) | Coverage >=10X  (%) | Coverage >=20X  (%) |
| --- | --- | --- | --- | --- |
| N4 | 13.31 | 88.73 | 49.76 | 5.56 |
| XiaF | 14.42 | 85.03 | 52.28 | 7.93 |
| AK | 12.83 | 80.70 | 43.12 | 5.06 |
| NanC | 7.76 | 66.62 | 14.53 | 0.45 |
