# Supplementary figures and images for: Copy number variations among silkworms
Source: BMC Genomics. 2014 Mar 31;15:251. doi: 10.1186/1471-2164-15-251 (PMC3997817; doi:10.1186/1471-2164-15-251)

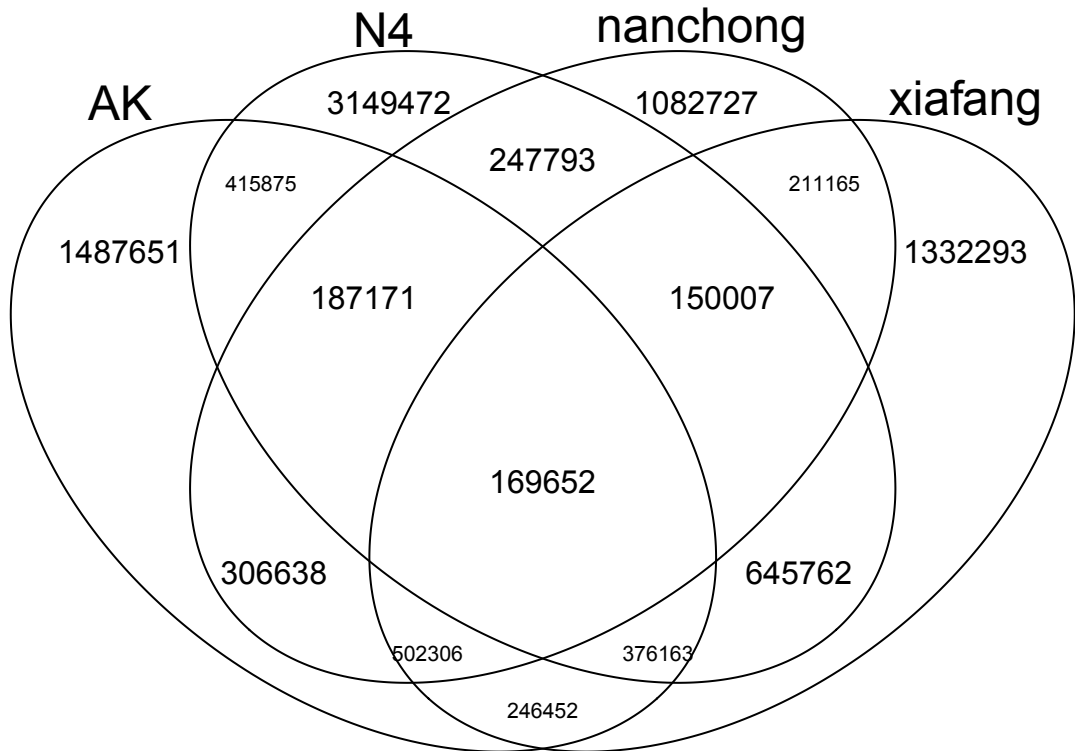

Supplement: Additional file 6 — Venn diagram showed the comparison of CNV content amongst different silkworms. [file 1471-2164-15-251-S6.pdf]

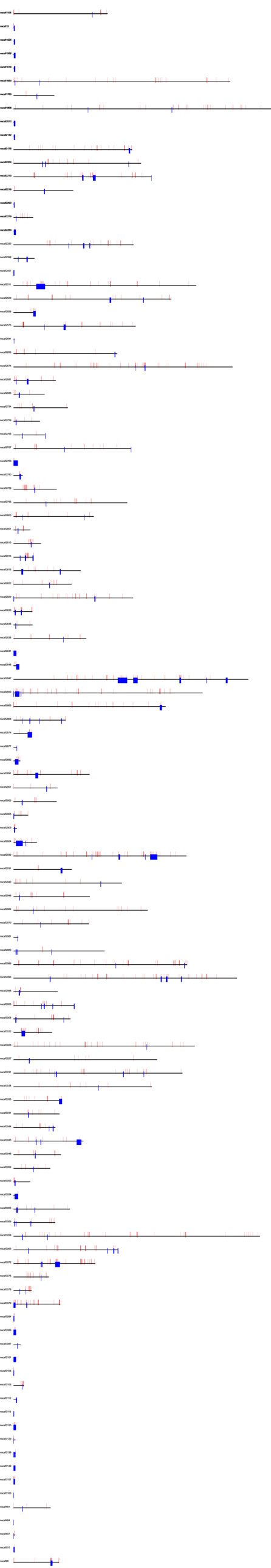

Supplement: Additional file 7 — Silkworm CNVs map. The silkworm assembly scaffold is represented as black bars. Larger bars in colors which intersect the scaffold represent the segmental duplications and copy number variation. [file 1471-2164-15-251-S7.pdf]

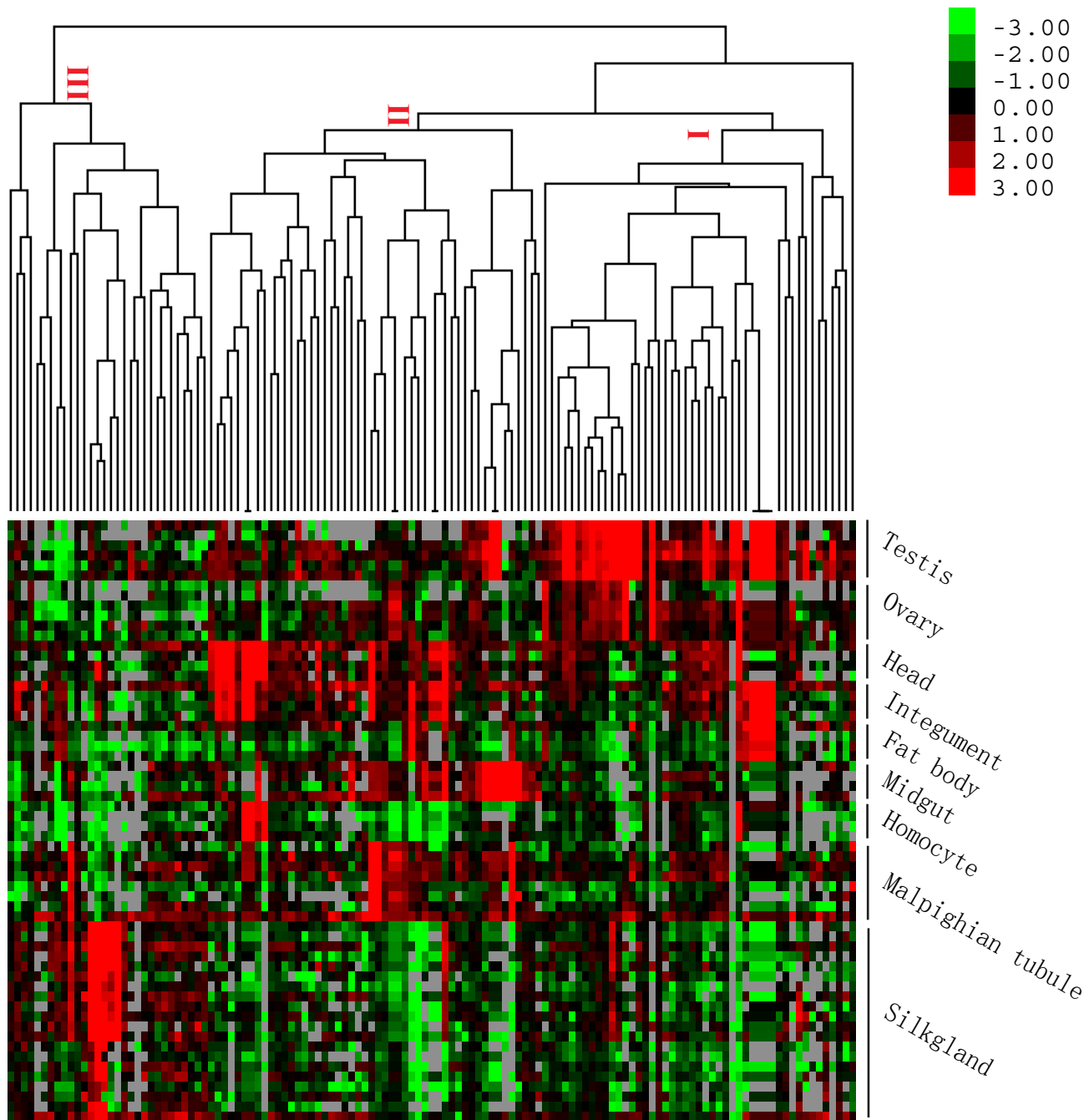

Supplement: Additional file 8 — Expression profiles of the genes located in CNVs based on microarray data. Hierarchical clustering with the average linkage method was performed. There were as many as 9 tissues used in the gene expression profiling. [file 1471-2164-15-251-S8.pdf]

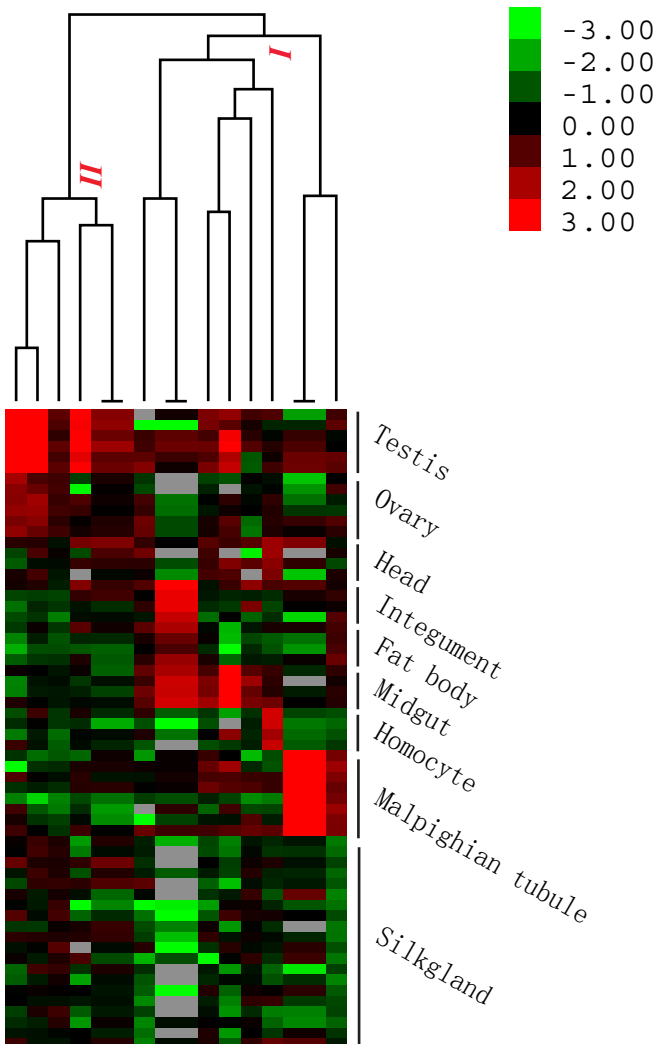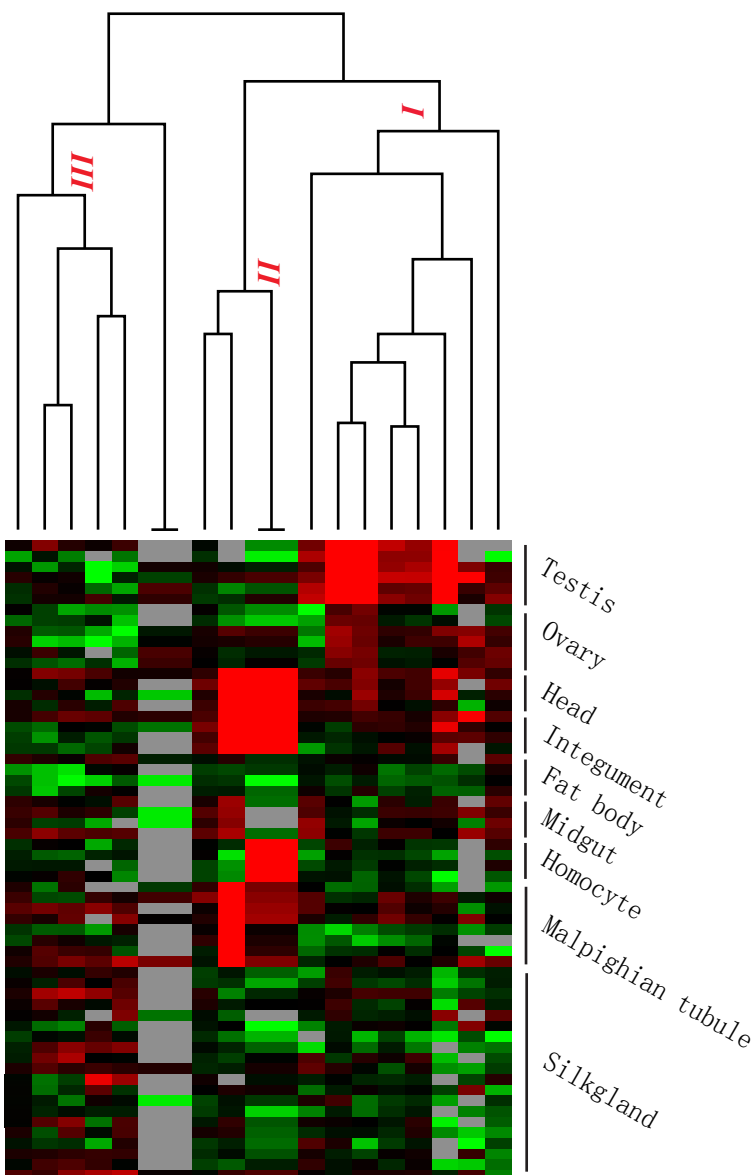

Supplement: Additional file 10 — Comparison of gene expression pattern located in domesticated-specific CNV regions and wild-specific CNVs based on microarray data. Hierarchical clustering with the average linkage method was performed. There were as many as 9 tissues used in the gene expression profiling. The upper diagram showed the expression profiles of genes in wild-specific CNVs. [file 1471-2164-15-251-S10.pdf]
