# Supplementary material for: Copy number variations among silkworms
Source: BMC Genomics. 2014 Mar 31;15:251. doi: 10.1186/1471-2164-15-251 (PMC3997817; doi:10.1186/1471-2164-15-251)

Additional 9. qPCR validation of predicted CNVs in silkworm and qPCR validation of

| Name | Location | Category | CNVs verified by qPCR |
| --- | --- | --- | --- |
| Target_r1 | scaffold984:1..11044 | loss | - |
| Target_r2 | scaffold956:1..12784 | loss | + |
| Target_r3 | scaffold880:1..15850 | Gain | + |
| Target_r4 | scaffold886:1..16003 | loss | + |
| Target_r5 | scaffold547:1..183479 | loss | + |

The other four genes validated by qPCR.


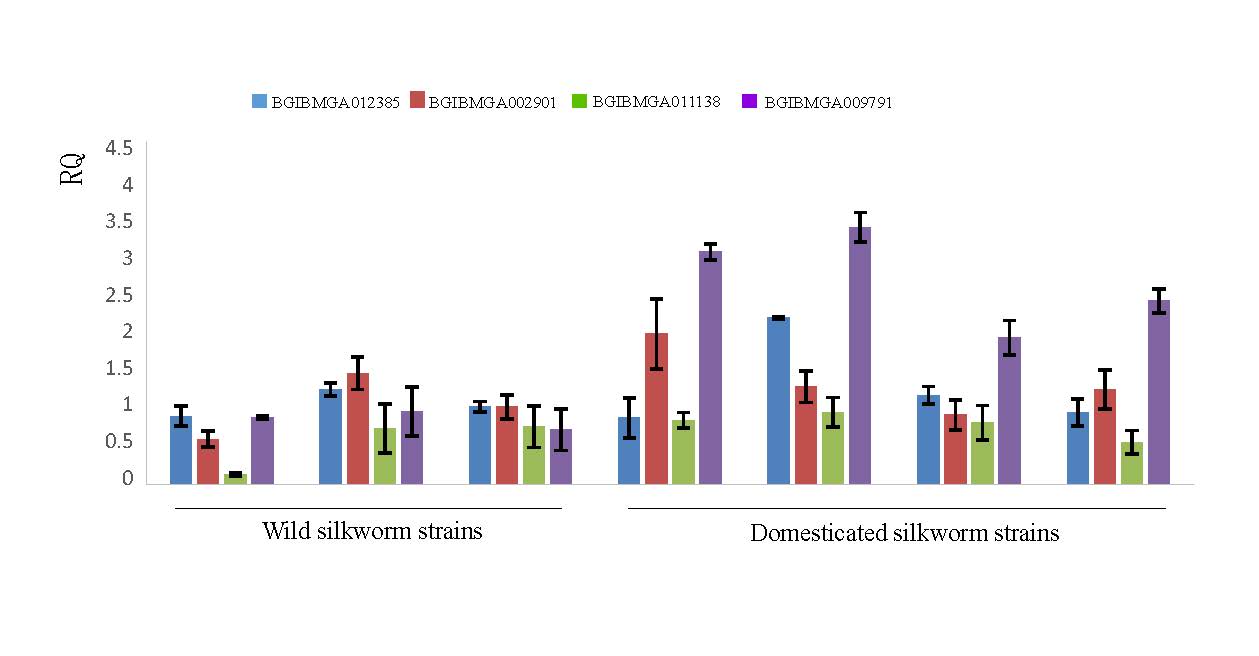

Supplement: Additional file 11 — qPCR validation of predicted CNVs in silkworms. [file 1471-2164-15-251-S11.doc]
