# Supplementary material for: Copy number variations among silkworms
Source: BMC Genomics. 2014 Mar 31;15:251. doi: 10.1186/1471-2164-15-251 (PMC3997817; doi:10.1186/1471-2164-15-251)
Supplement: Additional file 12 — Expression profiles of 8 genes in silkworm challenged by four pathogens: Bacillus bombyseptieus (BB, gram-positive bacteria); Beauveria bassiana (BJ, fungus); Escherichia coli (EC, gram-negative bacteria); B. mori Nuclear polyhedrosis viruses (NPV, virus). Data were collected from four time points (3 h, 6 h, 12 h and 24 h; for Be. bassinan: 6 h, 12 h, 24 h and 48 h) (Huang, 2010). [file 1471-2164-15-251-S12.pdf]

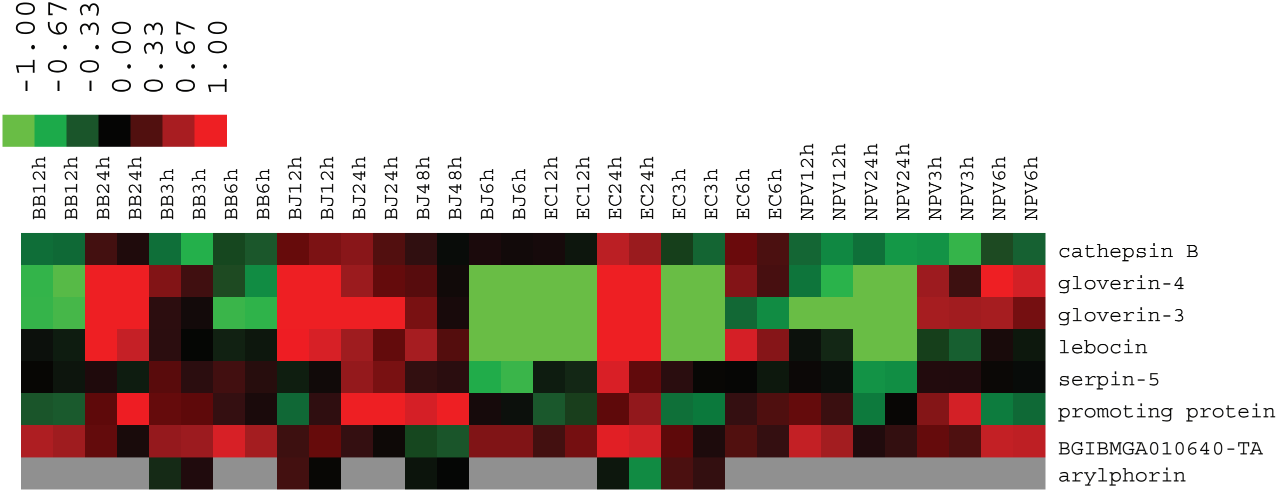

-1.00
-0.67
-0.33
0.00
0.33
0.67
1.00
BB12h
BB12h
BB24h
BB24h
BB3h
BB3h
BB6h
BB6h
BJ12h
BJ12h
BJ24h
BJ24h
BJ48h
BJ48h
BJ6h
BJ6h
EC12h
EC12h
EC24h
EC24h
EC3h
EC3h
EC6h
EC6h
NPV12h
NPV12h
NPV24h
NPV24h
NPV3h
NPV3h
NPV6h
NPV6h
cathepsin B
gloverin-4
gloverin-3
lebocin
serpin-5
promoting protein
BGIBMGA010640-TA
arylphorin
