# Supplementary material for: Copy number variations among silkworms
Source: BMC Genomics. 2014 Mar 31;15:251. doi: 10.1186/1471-2164-15-251 (PMC3997817; doi:10.1186/1471-2164-15-251)
Supplement: Additional file 13 — Thresholds for copy number gain and loss. [file 1471-2164-15-251-S13.doc]

**Additional file 14: Thresholds for copy number gain and loss.**

| Strain | N4 | XiaF | NanC | AK |
| --- | --- | --- | --- | --- |
| Loss | 1.342 | 1.346 | 1.351 | 1.345 |
| gain | 2.672 | 2.665 | 2.658 | 2.664 |
