# Supplementary material for: Copy number variations among silkworms
Source: BMC Genomics. 2014 Mar 31;15:251. doi: 10.1186/1471-2164-15-251 (PMC3997817; doi:10.1186/1471-2164-15-251)
Supplement: Additional file 14 — A list of primers used in qPCR. [file 1471-2164-15-251-S14.doc]

Additional file 11: Primer lists used in qPCR.

| **accession number** | **Primer Sequence** | ***T*_M_(℃)** |
| --- | --- | --- |
| OR2 | GCTGGATGTCATTTTCTGTTCGTGG | 61.98 |
|  | AACTCGGAACAACTCAGCCGTATTG | 61.98 |
| BGIBMGA010641 | GACATCCCTTACATCAGCA | 55.4 |
|  | ATACACCCGCAGGCATT | 54.6 |
| BGIBMGA014051 | TCCTCACTTCCACCACG | 57.01 |
|  | ACCGCTCCAAGAACCTG | 57.01 |
| BGIBMGA014052 | GACGGCTGCGATTTGTT | 54.59 |
|  | ATAGACGCTACCTCCTCCC | 53.2 |
| BGIBMGA014594 | AAATGTCTGCCGATAAACC | 53.3 |
|  | GCTGCCACTTGTCTCCTC | 52.7 |
| BGIBMGA014465 | ATTTGGCGATGACGACTC | 55.02 |
|  | TGTGGGTTCTTCCTGTGC | 57.3 |
| BGIBMGA014464 | ATTTGGCGATGACGACTC | 55.02 |
| BGIBMGA012385 | GCTTCACGCTCCATCAACT | 55.6 |
|  | GCGACGGAAACATTCTCAT | 55.6 |
| BGIBMGA002901 | TTTTCTGGAACGGGCTTTCAACTT | 65.6 |
|  | GTCAGCGTCTCGTCTCCGATGTAG | 65.1 |
| BGIBMGA011138 | TTTGTTGTACGGCTATTTCCTG | 57.7 |
|  | CTTCATTCCATACTCGCTGTCT | 56.5 |
| BGIBMGA009791 | GGGGACGAGGCATAATAACC | 58.6 |
|  | AACGCTGACTCCGCTACTGA | 58.3 |
|  | TGTGGGTTCTTCCTGTGC | 57.30 |
| Target_r1 | AGGTGTTTACTGGAACCCATAGA | 57.6 |
|  | AGCCGTTGTAACTATACTGAGACC | 57.1 |
| Target_r2 | ATCATCCAGAACATTCCACACA | 57.4 |
|  | GCCAACGACAAGGTAAGAGTAAC | 58.0 |
| Target_r3 | TTCCCCAACCTATTATGTCGC | 59.4 |
|  | CCCAGTTATGTGATTTTCAGAGC | 58.5 |
| Target_r4 | TATGGCGATGGTGATTATGCT | 58.2 |
|  | GCGAGAAACTCAGTAGTCTGTGTC | 57.8 |
| Target_r5 | CGTTCAAAGATTCCGTTACATAGAG | 59.8 |
|  | TTTACAAGGGTGGGGAGTTTAG | 58.2 |
| Con_R | CATGAAGATCCTCACCGAGCG |  |
|  | CGTAGCACAGCTTCTCCTTGATA |  |

***OR2*** was reported to be a single copy gene located on chromosome 16, while Con_R was a two-copy region (on copy on scaffold 2829 while another copy located on scaffold10921).
